# A Diverse and Versatile Regiospecific Synthesis of Tetrasubstituted Alkylsulfanylimidazoles as p38α Mitogen-Activated Protein Kinase Inhibitors

**DOI:** 10.3390/molecules23010221

**Published:** 2018-01-20

**Authors:** Francesco Ansideri, Stanislav Andreev, Annette Kuhn, Wolfgang Albrecht, Stefan A. Laufer, Pierre Koch

**Affiliations:** 1 Institute of Pharmaceutical Sciences, Department of Medicinal and Pharmaceutical Chemistry, Eberhard Karls Universität Tübingen, Auf der Morgenstelle 8, 72076 Tübingen, Germany; francesco.ansideri@uni-tuebingen.de (F.A.); stanislav.andreev@uni-tuebingen.de (S.A.); annette.kuhn@uni-tuebingen.de (A.K.); stefan.laufer@uni-tuebingen.de (S.A.L.); 2 Teva-ratiopharm, Graf-Arco-Str. 3, 89079 Ulm, Germany; wolfgang.albrecht@ratiopharm.de

**Keywords:** regiospecific synthesis, tetrasubstituted imidazoles, p38α MAP kinase

## Abstract

An alternative strategy for the synthesis of 1-aryl- and 1-alkyl-2-methylsulfanyl-4-(4-fluorophenyl)-5-(pyridin-4-yl)imidazoles as potential p38α mitogen-activated protein kinase inhibitors is reported. The regioselective *N*-substitution of the imidazole ring was achieved by treatment of α-aminoketones with different aryl or alkyl isothiocyanates. In contrast to previously published synthesis routes starting from 2-amino-4-methylpyridine, the presented route is characterized by a higher flexibility and a lower number of steps. This strategy was also applied to access 1-alkyl-2-methylsulfanyl-5-(4-fluorophenyl)-4-(pyridin-4-yl)imidazoles in six steps starting from 2-chloro-4-methylpyridine.

## 1. Introduction

The p38α mitogen-activated protein (MAP) kinase is a serine/threonine kinase, which plays a role in signal transduction pathways, modulating the cellular response to external stress stimuli like infection, heat or osmotic shock, UV light, and inflammatory cytokines [1]. Through phosphorylation of multiple downstream targets, this kinase triggers a wide range of cellular processes mostly resulting in the stimulation of the inflammatory reaction (e.g., release of pro-inflammatory cytokines like tumor necrosis factor-α, interleukin-1β, and interleukin-6 and induction of COX-2 transcription) [1,2]. The function of the p38α MAP kinase has therefore been regarded as crucial in those cytokine-driven chronical inflammatory conditions like rheumatoid arthritis, Crohn’s disease, psoriasis, and chronic asthma [3,4]. Additionally, several studies suggested that the p38α MAP kinase may also play a role as a major character in the pathogenesis of neurodegenerative diseases such as Alzheimer’s disease, Parkinson’s disease and multiple sclerosis [5,6,7,8]. Due to this well-documented physiopathological role, inhibition of the p38α MAP kinase has been widely pursued in many medicinal chemistry programs [9,10].

**Figure 1:**
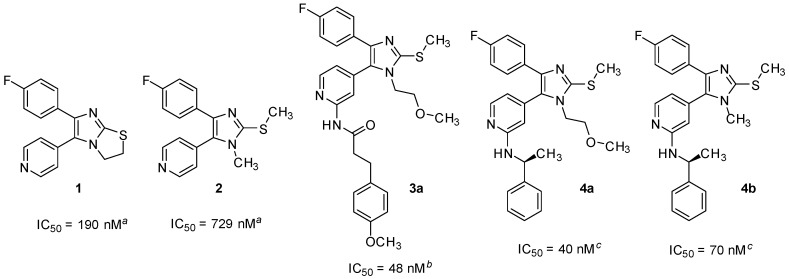
Early lead compound SKF86002 (1) and 1,2,4,5-tetrasubstituted imidazoles **2**, **3a**, **4a** and **4b** as p38α MAP kinase inhibitors. *^a^* unpublished data from our research group; compounds tested in assay conditions described by Goettert et al. [17]; *^b^* data taken from [14]; *^c^* data taken from [15].

Pyridinylimidazoles represent a privileged scaffold in the field of kinase inhibition, especially concerning the targeting of the p38α MAP kinase, as very recently reviewed by some of us [11]. In particular, tetrasubstituted pyridinylimidazoles like compounds **2**, **3a**, **4a** and **4b** [12,13,14,15] represent adenosine triphosphate (ATP)-competitive inhibitors of p38α MAP kinase, which might be considered as open analogues of the imidazolthiazolidine-based early lead compound SKF86002 (**1**) from SmithKline & French [16] (Figure 1).

**Figure 2:**
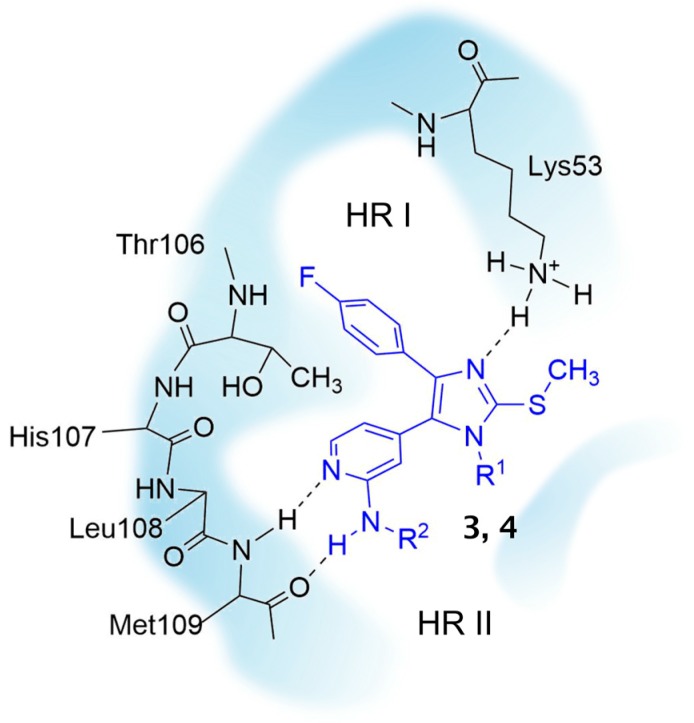
Binding mode of tetrasubstituted imidazoles bearing an acylamino or alkylamino group at the pyridine-C2 position. Figure modified from ref. [19].

Figure 2 shows the well-described binding mode of tetrasubstituted pyridinylimidazoles like compounds **2**, **3a**, **4a** and **4b** within the ATP cleft of the p38α MAP kinase. Worth to mention are the occupation of two hydrophobic pockets named hydrophobic region (HR) I and HR II as well as the formation of a hydrogen bond with the conserved Lys53 side chain. Tetrasubstituted pyridinylimidazoles were initially reported to display a lower inhibitory activity in comparison to the analogous trisubstituted derivatives lacking the substituent on the imidazole-N1 atom [18]. Nevertheless, it was observed that by inserting opportune substituents at this position, a high potency could still be maintained, probably thanks to additional interactions. Furthermore, some tetrasubstituted pyridinylimidazoles showed a markedly reduced inhibition of the CYP450 enzymes, considered one of the major drawbacks of their trisubstituted counterparts [13].

Due to the binding mode of this class of inhibitors, a compulsory structural requisite is that the substituted imidazole-N atom is the one adjacent to the pyridine ring. Substitution on the imidazole-N atom proximal to the 4-fluorophenyl ring would instead prevent the formation of the hydrogen bond with Lys53 and has been reported to cause a tremendous drop in inhibitory activity [20]. For this reason, in order to preserve their binding affinity to the p38α MAP kinase, these inhibitors need to be accessed through a regiospecific synthetic route.

**Scheme 1:**
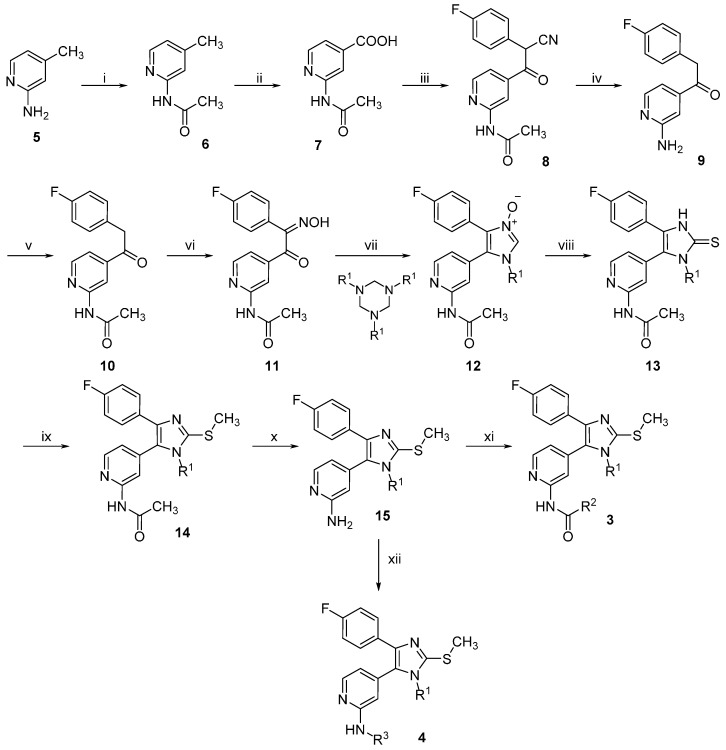
Synthetic pathway toward 1,2,4,5-tetrasubstituted imidazoles **3** and **4**. Reagents and conditions: (i) Ac_2_O, DMAP, reflux temperature; (ii) KMnO_4_, H_2_O, 50–90 °C; (iii) CDI, DMF, *t*-BuOK, 120 °C; (iv) HBr, reflux temperature; (v) Ac_2_O, DMAP, reflux temperature; (vi) isoamyl nitrite, MeONa, MeOH, rt; (vii) EtOH, reflux temperature; (viii) 2,2,4,4-tetramethylcyclobutane-1,3-dithione, DCM, rt; (ix) iodomethane, Na_2_CO_3_, EtOH, rt; (x) 10% HCl_(aq)_, reflux temperature; (xi) R^2^-COCl, NEt_3_, THF, 0 °C or R^2^-COOH, CDI, *N*-methylpyrrolidinone, rt, then 120 °C; (xii) R^3^-Br, NaH, DMF, rt.

The first synthetic route toward tetrasubstituted 2-alkylsulfanylimidazoles was reported in 2002 and 2003 (Scheme 1) [21,22] and was subsequently employed in several studies on kinase inhibitors [13,14,15,23,24,25]. This route, comprising eleven steps, starts from 2-amino-4-methylpyridine (**5**) which was first protected as an acetamide and successively oxidized to a carboxylic acid. Ethanone **9** was then obtained by coupling with 4-fluorophenylacetonitrile followed by hydrolysis-decarboxylation reaction of the resulting cyanoketone **8**, causing the simultaneous cleavage of the *N*-acetyl protecting group. After repeating the protection step, ethanone **10** was converted into the α-oximinoketone **11**, and then cyclized with an opportunely substituted 1,3,5-trialkyltriazinane to regioselectively afford imidazole-*N*-oxides **12**. Intermediates **12** were then transformed into the corresponding imidazole-2-thiones **13** and then methylated on the exocyclic sulphur. Finally, the amino group of compounds **14** was deprotected and then coupled with different carboxylic acids to produce final compounds **3**. From intermediates **15** it is also possible to obtain alkylamino derivatives **4** by nucleophilic substitution reaction with alkyl halides [13].

This reported route noticeably entails a large number of steps and the preparation of intermediate **10** is particularly inconvenient due to the repeated introduction and cleavage of the *N*-acetyl protecting group. An additional drawback of the reported route is the flexibility of application. As reported from the authors, this route is indeed not suitable for the introduction of aryl or heteroaryl moieties at the imidazole-N1 position [22]. Moreover, the functionalization of the pyridine-C2 position by nucleophilic substitution reaction of 2-aminopyridine derivatives **15** and alkyl halides represents a limitation when introducing alkyl groups featuring a stereocenter adjacent to the amino moiety. The use of chiral alkyl halides can indeed result in inversion of configuration or racemization, thus hampering the stereoselective synthesis of some derivatives (e.g., compound **4a**, wherein the hypothetical use of the corresponding benzyl halide would favor an S_N_1 mechanism with the consequent loss of chirality). For this reason, preparation of derivatives **4a** and **4b** could only be achieved by converting the 2-amino group into a fluorine atom and introducing the chiral amine by nucleophilic aromatic substitution, as reported in 2008 [12].

**Scheme 2:**
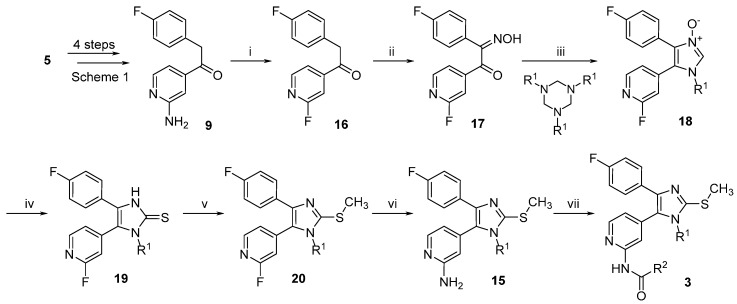
Synthetic pathway toward 1,2,4,5-tetrasubstituted imidazoles **3**. Reagents and conditions: (i) NaNO_2_, 70% HF-pyridine; −15 to −10 °C, then rt; (ii) NaNO_2_, glacial AcOH, rt; (iii) EtOH, reflux temperature; (iv) 2,2,4,4-tetramethylcyclobutane-1,3-dithione, DCM, rt; (v) iodomethane, K_2_CO_3_, MeOH, rt; (vi) NH_3_, reactor; (vii) R^2^-COOH, CDI, *N*-methylpyrrolidinone, rt, then 120 °C.

In 2011, Selig et al. published an alternative synthetic route to tetrasubstituted imidazoles **3** (Scheme 2) [18]. This route is overall similar to the previously reported one and only differs in the replacement of the amino group of intermediate **9** with a fluorine, allowing the realization of the following steps without the necessity of the protecting group. The amino group was then reintroduced at the penultimate step, before its functionalization with different acyl derivatives yielding compounds **3**. Nevertheless, because of the replacement of the amino group only after the preparation of ethanone **9**, this route does not bypass the issues relative to the installation and cleavage of the *N*-acetyl protecting group and encompasses the same number of steps as the one presented in Scheme 1.

## 2. Results and Discussion

### 2.1. Chemistry

**Scheme 3:**
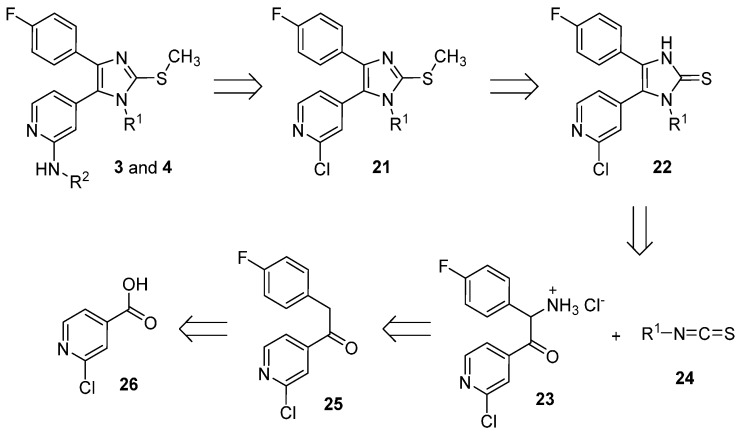
Retrosynthetic strategy for the preparation of 1,2,4,5-tetrasubstituted imidazoles **3** and **4**.

Our retrosynthetically-planned synthetic strategy towards 1,2,4,5-tetrasubstituted imidazoles **3** and **4** is depicted in Scheme 3. In contrast to the aforementioned routes, we sought to introduce the whole alkyl- or acylamino group at the pyridine-C2 position starting from 2-chloropyridine derivatives **21** since the higher stability of the 2-halopyridine moiety avoids the need for protection/deprotection steps. However, in contrast to the route reported by Selig et al., chlorine was preferred over fluorine, as it can be displaced through both nucleophilic aromatic substitution reaction and Buchwald-Hartwig arylamination/amidation. Moreover, this choice would allow the introduction of chiral amines having the stereocenter adjacent to the amino group without leading to racemization or inversion of configuration, as the reaction mechanism does not involve the chiral center. Key passage of the synthesis is then the cyclization reaction of α-aminoketone **23** and alkyl/aryl-substituted isothiocyanates **24**, allowing the regioselective introduction of the imidazole-N-substituent, followed by methylation of resulting intermediates **22**. Finally, a suitable precursor for the preparation of α-aminoketone **23** could be represented by ethanone derivative **25**, which in turn can arise from the commercially available 2-chloroisonicotinic acid (**26**).

**Scheme 4:**
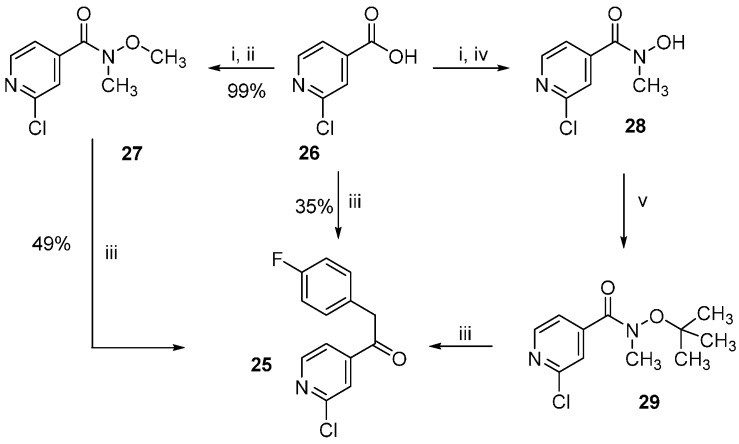
Diverse strategies for the synthesis of ethanone derivative **25**. Reagents and conditions: (i) SOCl_2_, reflux temperature; (ii) *N*,*O*-dimethylhydroxylamine hydrochloride, NEt_3_, DCM, rt; (iii) 4-fluorobenzyl magnesium chloride, dry THF, −10 °C, then rt; (iv) *N*-methylhydroxylamine hydrochloride, NEt_3_, dry DCM, rt; (v) *t*-BuOAc, 70% HClO_4(aq)_, 1,4-dioxane, 60 °C, sealed vial.

The initial step of our alternative route consisted in the preparation of the 1-(2-chloropyridin-4-yl)-2-(4-fluorophenyl)ethan-1-one (**25**). This compound represents the 2-chloropyridine-substituted analogue of derivative **9**, a common intermediate of both published synthetic pathways toward tetrasubstituted pyridinylimidazoles (Scheme 1 and Scheme 2). As already mentioned, the preparation of ethanone **9** constituted one of the major bottlenecks of both previously reported routes and as a consequence, the new synthetic strategy would benefit from a more efficient method to achieve its analogue **25**. Several attempts were carried out in order to yield compound **25**, which are displayed in Scheme 4.

Despite their similarity, it was not possible to prepare compound **25** in an analogous fashion as ethanone **9**, namely by condensation of the 2-chloroisonicotinic acid with 4-fluorophenylacetonitrile followed by hydrolysis of the resulting cyanoketone. A convenient method for the synthesis of ketones is generally represented by the addition of Grignard reagents to Weinreb amides. Unfortunately, Grignard reaction of 4-fluorobenzyl magnesium chloride with 2-chloro-*N*-methoxy-*N*-methylisonicotinamide (**27**) afforded ethanone **25** in a non-satisfactory yield of 49%. A reason for the reduced yield is probably a well-described E_2_ elimination reaction starting with the extraction of a proton from the *N*-methoxy group and resulting in the formation of the corresponding *N*-methylamide and formaldehyde [26]. In order to avoid this undesired side reaction a previously described approach was followed [27], consisting in the use of a modified Weinreb amide wherein the methoxy group was replaced by a *tert*-butoxy moiety. Such group has no H atoms adjacent to the oxygen atom and is therefore not prone to deprotonation by the Grignard reagent. *N*-(*tert*-butoxy)-2-chloro-*N*-methylisonicotinamide (**29**) could be prepared in good yield via a two-step procedure starting from the corresponding carboxylic acid **26**. In detail, coupling of **26** with *N*-methylhydroxylamine afforded the corresponding hydroxamic acid **28**, which was then converted into the Weinreb amide **29** by acid-catalyzed esterification with *tert*-butyl acetate. The use of amide **29** in the Grignard reaction with 4-fluorobenzyl magnesium chloride permitted a significantly increased yield of up to 81%, with an overall yield of 62% over 3 steps for the preparation of intermediate **25**. It is worth mentioning that the ethanone derivative **25** could also be prepared by direct Grignard reaction of 2-chloroisonicotinic acid (**26**) with 4-fluorobenzyl magnesium chloride, adapting a procedure previously reported by Reeves et al. [28]. The latter method allows the preparation of key intermediate **25** in a single step and consists in reacting the carboxylic acid with two equivalents of organomagnesium reactant in order to achieve nucleophilic addition on the carboxylate derivative. This approach afforded derivative **25** in a low yield (35%) and the formation of 1,2-bis(4-fluorophenyl)ethane as a by-product was observed.

**Scheme 5:**
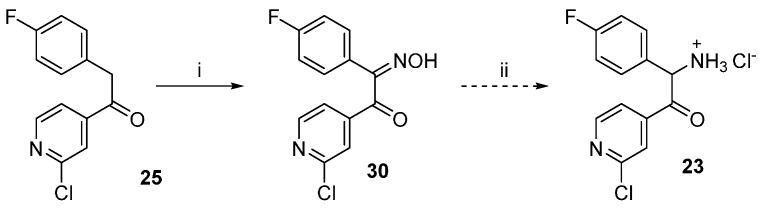
Attemped short strategy for the preparation of α-aminoketone **23**. Reagents and conditions: (i) NaNO_2_, glacial AcOH, rt; (ii) Pd/C 10%, H_2_, isopropanolic HCl, rt.

**Scheme 6:**
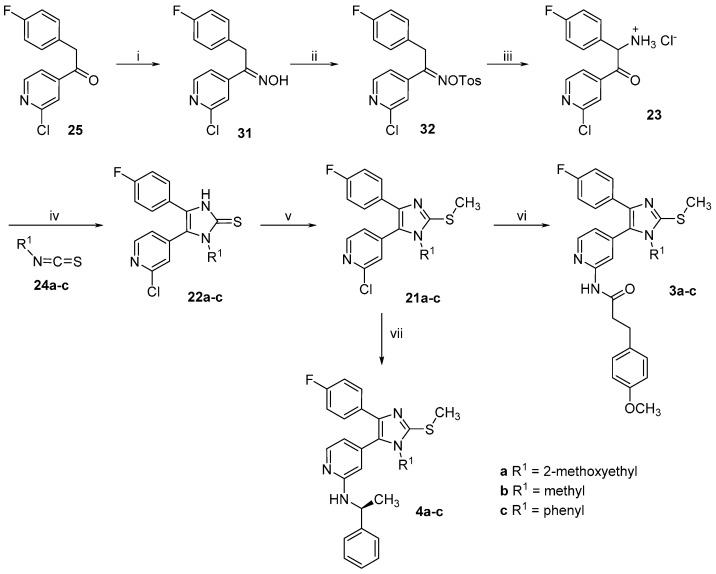
Optimized synthesis of tetrasubstituted imidazoles **3** and **4**. Reagents and conditions: (i) hydroxylamine hydrochloride, NaOH_(aq)_, MeOH, rt; (ii) tosyl chloride, pyridine, rt; (iii) (a) EtOK, EtOH_(abs)_, 0 °C, then rt; (b) conc. HCl_(aq)_, 50 °C; (iv) (a) NEt_3_, 50 °C; (b) glacial AcOH, 80 °C; (v) iodomethane, *t*-BuONa, MeOH, 50 °C; (vi) 3-(4-methoxyphenyl)propanamide, Pd_2_(dba)_3_, XantPhos, Cs_2_CO_3_, DMF, 100 °C; (vii) (*S*)-phenylethan-1-amine, 180 °C, sealed tube.

After obtaining a convenient method for the preparation of ethanone intermediate **25**, a strategy for its conversion into α-aminoketone **23** was pursued. As shown in Scheme 5, reduction of the α-ketoxime **30** did not succeed in producing the desired α-aminoketone **23** starting from ethanone **25**. Such transformation was instead successfully achieved through a three-step procedure involving a base-mediated Neber rearrangement of an *O*-tosyl-oxime derivative (Scheme 6). In detail, ethanone **25** was first reacted in a nucleophilic addition with hydroxylamine and the resulting oxime **31** was tosylated. Following the tosylation protocol of Lantos and coworkers for a related ketoxime [29], a significantly slower conversion was observed, accompanied by the formation of by-products, which deteriorated under increased thermal conditions. Carrying out the reaction at room temperature afforded a sufficiently clean tosylation, yet requiring a high excess of *p*-toluenesulfonylchloride to compensate for the resultant low conversion rate. Intermediate **32** was then reacted in a Neber rearrangement with potassium ethoxide, affording an aziridine derivative, which was immediately hydrolyzed in acidic conditions, yielding α-aminoketone **23** as a hydrochloride salt.

Construction of the imidazole ring was performed by reaction of the α-aminoketone **23** and alkyl or aryl isothiocyanates **24** in a single two-step procedure. First, the nucleophilic addition of the amino group to the isothiocyanate took place using triethylamine as both base and solvent. The cyclization was then promoted by evaporating the triethylamine and by heating the reaction mixture in glacial acetic acid, succeeding in the regioselective preparation of *N*-alkyl and -aryl substituted imidazole-2-thiones **22** in moderate yields. Tetrasubstituted imidazole derivatives **21** were then easily accessible through nucleophilic substitution of compounds **22** with iodomethane in the presence of a base. The last step of our alternative route consisted in the introduction of the alkyl- or acylamino functions at the pyridine-C2 position. In case of aliphatic amines this was obtained by nucleophilic aromatic substitution of synthones **21** with large excess of amine in solvent-free conditions. Amide moieties could instead be introduced starting from the same derivative via palladium-catalyzed Buchwald-Hartwig reaction.

**Scheme 7:**
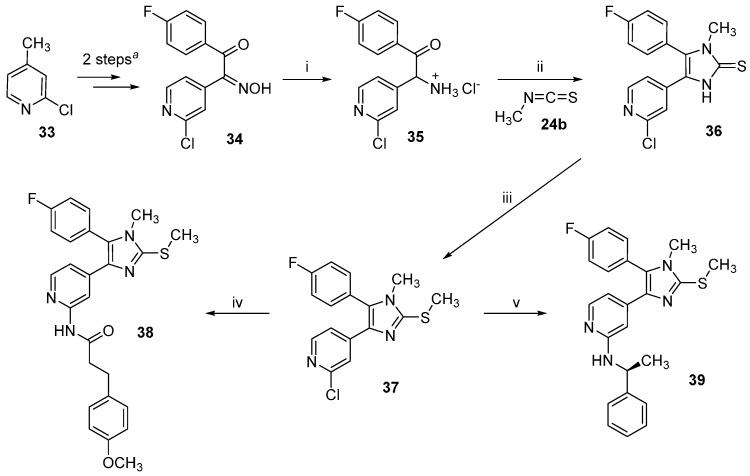
Synthesis of tetrasubstituted imidazoles **38** and **39**. ^a^ Procedure reported by Laufer et al. [30]. Reagents and conditions: (i) Pd/C 10%, H_2_, isopropanolic HCl, rt; (ii) (a) NEt_3_, 60 °C; (b) glacial AcOH, 80 °C; (iii) iodomethane, *t*-BuONa, MeOH, 50 °C; (iv) 3-(4-methoxyphenyl)propanamide, Pd_2_(dba)_3_, XantPhos, Cs_2_CO_3_, DMF, 100 °C; (v) (*S*)-1-phenylethan-1-amine, 180 °C, sealed tube.

As already mentioned, derivatization of the imidazole-N atom adjacent to the 4-fluorophenyl ring is detrimental for inhibitory activity on the p38α MAPK, as the introduced alkyl or aryl substituent prevents the formation of a hydrogen bond interaction with the Lys53 of the enzyme. Nevertheless, in order to broaden the applicability of our alternative synthetic pathway, we tested its suitability for the regioselective synthesis of 1,2,4,5-tetrasubstituted pyridinylimidazoles bearing the substituent on the imidazole-N atom distal from the pyridine ring (Scheme 7). In this series, derivatives **38** and **39**, bearing a methyl group on the imidazole-N atom, were prepared as an example to prove the applicability of the presented synthetic route. In order to invert the substitution pattern on the imidazole ring, the cyclization step clearly needs to be performed starting from α-aminoketone **36**, a regioisomer of compound **23**. This derivative could be obtained via a previously described procedure consisting in the condensation of 2-chloro-4-picoline (**33**) with ethyl 4-fluorobenzoate followed by α-nitrosylation of the ethanone intermediate **34** and reduction of the resulting α-ketoxime **34**. Differently from the attempt depicted in Scheme 4, the α-ketoxime **34**, presenting the opposite arrangement of the two aryl substituents, could be smoothly reduced affording aminoketone **35** in very good yields. Starting from the α-aminoketone **35,** the cyclization-methylation steps were carried out in an analogous fashion as the ones described in Scheme 6. Finally, using the same conditions as for compounds **3** and **4**, compounds **38** and **39** could be obtained by Buchwald-Hartwig amidation and by nucleophilic aromatic substitution, respectively.

### 2.2. Biological Evaluation and SAR Insights

Compounds **3b**–**c**, **4c**, **38** and **39** were tested in an enzyme-linked immunosorbent assay (ELISA) in order to evaluate their capability to inhibit the p38α MAP kinase and the results were compared to the ones of compounds **3a** and **4a**–**b**, which were previously tested in the same assay [17] (Table 1). Although the scant number of evaluated compounds does not allow an exhaustive analysis of structure-activity relationships, tetrasubstituted pyridinylimidazoles were confirmed as potent inhibitors of the p38α MAP kinase, reaching IC_50_ values down to the low double-digit nanomolar range (compound **3b**). As expected, substitution of the N atom adjacent to the 4-fluorophenyl ring (compounds **38** and **39**) was detrimental for the inhibitory activity, due to the suppression of the hydrogen bond with the Lys53. When comparing the two compound series **3a**–**c** and **4a**–**c** it appears evident that substitution of the imidazole-N atom with a phenyl ring (compounds **3c** and **4c**) has a negative effect on the inhibitory activity, even reaching an IC_50_ value in the micromolar range in case of compound **4c**. This can be either due to unfavorable interactions with the phosphate/sugar pocket or to a sterical hindrance with the 2-alkylamino- or 2-acylaminopyridine moiety, which does not allow a correct positioning of the molecule in the binding pocket of the enzyme. Finally, whereas the acylamino substituent appears to be overall preferable to the alkylamino group at the pyridine-C2 position, no clear indication could be obtained regarding the superiority of either the methyl or the methoxyethyl substituent at the imidazole-N atom.

To sum up, the herein reported route represents a valid alternative for the synthesis of tetrasubstituted pyridinylimidazoles, a class of molecules counting several examples in the field of kinase inhibition due to the capability of reaching high inhibitory potency together with reduced interaction with the CYP450 enzymes. This route comprises a lower number of synthetic steps in comparison with previously reported strategies along with an increased versatility. Both aliphatic and aromatic moieties can be introduced at the imidazole-N1 atom without modifying the synthetic path. Furthermore, the range of possible substituents is extremely broad thanks to both the commercial availability of diversely substituted isothiocyanates and to reported procedures describing facile preparation methods for these intermediates [31,32,33]. The presence of a Cl atom at the pyridine-C2 position eliminates the necessity of protection/deprotection steps and permits the functionalization with both amines and amides in the last step of the route. Furthermore, chiral amines featuring the stereocenter in the α-position can be introduced without the risk of inversion of configuration or racemization.

The introduction of an aromatic ring on the imidazole-N atom, constituting one of the advantages of the presented route with respect to published ones, did not emerge as a beneficial substitution to increase the inhibitory activity on the p38α MAP kinase; likewise, functionalization of the imidazole-N atom distal to the pyridine ring results in a significantly reduced potency on the same target. Nevertheless, since pyridinylimidazoles represent a privileged scaffold in the realm of kinase inhibition, these synthetic improvements can still result helpful in the targeting of different kinases having dissimilar structural features compared to the p38α MAP kinase.

## 3. Materials and Methods 

### 3.1. General Information

All reagents and solvents were of commercial quality and utilized without further purification. Thin layer chromatography (TLC) reaction controls were performed for all reactions using fluorescent silica gel 60 F_254_ plates (Merck, Darmstadt, Germany) and visualized under natural light and UV illumination at 254 and 366 nm. The purity of all tested compounds are >95% as determined via reverse phase high performance liquid chromatography (HPLC) on a 1100 Series HPLC system (Agilent, Santa Clara, CA, USA) equipped with a UV diode array detector (detection at 218 nm, 254 nm and 280 nm). The chromatographic separation was performed on a XBridge™ C18 column (150 mm × 4.6 mm, 5 µm) at 24 °C oven temperature. The injection volume was 10 μL and the flow was 1.5 mL/min using the following gradient: 0.01 M KH_2_PO_4_, pH 2.3 (solvent A), MeOH (solvent B), 45% B to 85% B in 10 min; 85% B for 6 min; stop time 16 min. Column chromatography was performed on Davisil LC60A 20–45 µm silica from Grace Davison (Columbia, MD, USA) and Geduran Si60 63–200 µm silica from Merck for the pre-column using an PuriFlash 430 automated flash chromatography system (Interchim, Montluçon, France). Nuclear magnetic resonance (NMR) spectra were measured at 300/75 MHz on an Avance III HD NMR spectrometer (Bruker, Billerica, MA, USA) at the Organic Chemistry Institute, Eberhard Karls Universität Tübingen. Chemical shifts are reported in parts per million (ppm) relative to tetramethylsilane. All spectra were calibrated against the (residual proton) peak of the deuterated solvent used. Mass spectra were performed on an Expression S electrospray ionization mass spectrometer (ESI-MS, Advion, Ithaca, NY, USA) with TLC interface in the Institute of Pharmaceutical Sciences, Eberhard Karls Universität Tübingen. High-resolution mass spectra (HRMS) were measured on a Bruker maXis 4G ESI time of flight mass spectrometer (ESI-TOF-MS) in the positive mode in the Organic Chemistry Institute, Eberhard Karls Universität Tübingen.

### 3.2. Experimental Procedures

#### 3.2.1. General Procedure for the Preparation of Imidazole-2-Thione Derivatives **22a–c** and **36** (General Procedure A)

In a pressure vial the corresponding alkyl or aryl isothiocyanate **24** (3–5 equiv.) was dissolved in NEt_3_ (2 mL) and then the appropriate α-aminoketone derivative **35** or **23** (1 equiv.) was added. The closed vial was heated at 50 °C and stirred until the starting compound resulted completely consumed as detected by HPLC analysis (1.5–4 h). The NEt_3_ was then evaporated at reduced pressure and the residue was taken up in glacial AcOH and stirred at 80 °C for 2 to 16 h. After concentrating the mixture at reduced pressure, a NaHCO_3_ saturated solution was added until reaching pH ≈ 8 and the aqueous layer was then extracted with EtOAc. The combined organic layers were then washed with NaCl saturated solution, dried over anhydrous Na_2_SO_4_, and concentrated at reduced pressure. The residue was finally purified by flash column chromatography.

#### 3.2.2. General Procedure for the Preparation of 2-Methylsulfanylimidazole Derivatives **21a–c** and **37** (General Procedure B)

In a pressure vial the appropriate imidazole-2-thione derivative (**22a**–**c** or **36**, 1 equiv.) was suspended in MeOH (4–10 mL) and after that *t*-BuONa (1.2 equiv.) was added. After cooling the mixture at 0 °C, iodomethane (5 equiv.) was added and the tightly closed vial was heated to 50 °C and stirred for 45 min to 2 h. After removing the solvent at reduced pressure H_2_O was added and the aqueous phase was extracted with DCM. The combined organic layers were then washed with NaCl saturated solution, dried over anhydrous Na_2_SO_4_, and concentrated at reduced pressure. The residue was finally purified by flash column chromatography or directly used for the following step.

#### 3.2.3. General Procedure for the Preparation of Amides **3a–c** and **38** (General Procedure C)

Under argon atmosphere the corresponding imidazole derivative **21a**–**c** or **37** (1 equiv.), 3-(4-methoxyphenyl)propanamide (1.5 equiv.), Pd_2_(dba)_3_ (0.05 equiv.), XantPhos (0.1 equiv.), and Cs_2_CO_3_ (3 equiv.) were suspended in dry DMF and after that the reaction mixture was heated at 100 °C and stirred overnight (18 h). The reaction mixture was poured in H_2_O and the aqueous phase was extracted with EtOAc. The combined organic layers were then washed with NaCl saturated solution, dried over anhydrous Na_2_SO_4_, and concentrated at reduced pressure. The residue was finally purified by flash column chromatography.

#### 3.2.4. General Procedure for the Preparation of Amines **4a–c** and **39** (General Procedure D)

In a pressure vial, the corresponding imidazole derivative **21a**–**c** or **37** (1 equiv.) was suspended in (*S*)-1-phenylethan-1-amine (1.5 mL) and the closed vial was stirred at 180 °C for 18–40 h. The reaction mixture was poured in H_2_O and the aqueous phase was extracted with EtOAc. The combined organic layers were then washed with NaCl saturated solution, dried over anhydrous Na_2_SO_4_, and concentrated at reduced pressure. The residue was finally purified by flash column chromatography.

#### 3.2.5. Detailed Procedures for the Preparation of Synthesized Compounds

*2-Chloro-N-methoxy-N-methylisonicotinamide* (**27**). 2-Chloroisonicotinic acid (**26**, 21.0 g, 133.3 mmol) was suspended in SOCl_2_ (60 mL) and the mixture was stirred at reflux temperature for 5 h. After removing the excess of solvent, the residue was taken up in dry DCM and added dropwise to an ice-cooled previously prepared suspension of *N*,*O*-dimethylhydroxylamine hydrochloride (15.6 g, 160 mmol) and NEt_3_ (45.0 mL, 320 mmol) in dry DCM (50 mL). After completion of the addition the mixture was let heating at rt and stirred overnight. After evaporating the solvent at reduced pressure H_2_O was added and the aqueous phase was extracted with DCM. The combined organic layers were then washed with NaCl saturated solution, dried over anhydrous Na_2_SO_4_, and concentrated at reduced pressure giving 26.0 g of product as a light brown solid, which was directly used for the following step without further purification (97% yield); ^1^H-NMR (300 MHz, CDCl_3_) δ 3.35 (s, 3H), 3.53 (s, 3H), 7.43 (dd, *J* = 5.0, 1.2 Hz, 1H), 7.54 (s, 1H), 8.45 (d, *J* = 5.1 Hz, 1H); ^13^C-NMR (75 MHz, CDCl_3_) δ 32.9, 61.5, 120.7, 122.8, 144.5, 149.8, 151.6, 166.0; ESI-MS: (*m*/*z*) 201.1 [M + H]^+^; HPLC: t_r_ = 2.051 min.

*2-Chloro-N-hydroxy-N-methylisonicotinamide* (**28**)*.* 2-Chloroisonicotinic acid (**26**, 5.0 g, 31.7 mmol) was suspended in SOCl_2_ (25 mL) and the mixture was stirred at reflux temperature for 6 h. After removing the excess of solvent, the residue was taken up in dry DCM and added dropwise to an ice-cooled previously prepared suspension of *N*-methylhydroxylamine hydrochloride (3.2 g, 38.0 mmol) and NEt_3_ (9.7 mL, 76.1 mmol) in dry DCM (15 mL). After completion of the addition the mixture was let heating at rt and stirred overnight. After evaporating the solvent at reduced pressure H_2_O was added and the aqueous phase was extracted with EtOAc. The combined organic layers were then washed with NaCl saturated solution, dried over anhydrous Na_2_SO_4_, and concentrated at reduced pressure giving 5.6 g of product as a white-pink solid, which was directly used for the following step without further purification (95% yield); ^1^H-NMR (300 MHz, DMSO-*d*_6_) δ 3.27 (s, 3H), 7.53 (dd, *J* = 5.0, 1.2 Hz, 1H), 7.61 (dd, *J* = 1.2, 0.5 Hz, 1H), 8.49 (dd, *J* = 5.0, 0.5 Hz, 1H), 10.40 (br. s, 1H); ^13^C-NMR (75 MHz, DMSO-*d*_6_) δ 36.9, 122.1, 123.1, 146.5, 150.50, 150.56, 165.7; ESI-MS: (*m*/*z*) 187.2 [M + H]^+^, 185.1 [M − H]^−^; HPLC: t_r_ = 1.568 min.

*N-(tert-butoxy)-2-chloro-N-methylisonicotinamide* (**29**). In a pressure vial 2-chloro-*N*-hydroxy-*N*-methylisonicotinamide (**27**, 500 mg, 2.68 mmol) was suspended in dry 1,4-dioxane (5 mL) and *t*-BuOAc (20 mL). After adding 70% HClO_4(aq)_ (27 mg, 0.27 mmol) the vial was tightly closed and the mixture was stirred at 60 °C for 48 h. After cooling down, the mixture was poured in a K_2_CO_3_ saturated solution and the aqueous phase was extracted with EtOAc. The combined organic layers were then dried over anhydrous Na_2_SO_4_ and the solvent was evaporated at reduced pressure giving 402 mg of the desired compound, which was used for the following step without further purification; ^1^H-NMR (300 MHz, CDCl_3_) δ 1.10 (s, 9H), 3.43 (s, 3H), 7.48 (dd, *J* = 5.1, 1.2 Hz, 1H), 7.60 (br. s, 1H), 8.43 (dd, *J* = 5.0, 0.5 Hz, 1H); ESI-MS: (*m*/*z*) 243.1 [M + H]^+^; HPLC: t_r_ = 4.733 min.

*1-(2-Chloropyridin-4-yl)-2-(4-fluorophenyl)ethan-1-one* (**25**). The title compound could be obtained alternatively through the following three procedures:

1) Under argon atmosphere Mg turnings (1.2 g, 50.0 mmol) were suspended in dry THF (120 mL) and after that 4-fluorobenzyl chloride (6.0 g, 41.5 mmol) was added in one portion. After the reaction was initiated, the mixture warmed up and was stirred until it cooled down to rt. The residual Mg was let decanting and the supernatant was added dropwise to a solution of 2-chloro-*N*-methoxy-*N*-methylisonicotinamide (**27**, 3.2 g, 16.0 mmol) in dry THF (35 mL) under argon atmosphere. The mixture was then stirred for 2 h at a temperature of 30–35 °C. The reaction was quenched with NH_4_Cl saturated solution (100 mL) and stirred overnight at rt. The two formed phases were separated and the aqueous phase was then further extracted by EtOAc. The combined organic layers were then washed with NaCl saturated solution, dried over anhydrous Na_2_SO_4_, and concentrated at reduced pressure. Finally, the residue was purified by flash column chromatography (SiO_2_, petroleum ether 40/60: EtOAc 4:1) giving 1.96 g of pure product as a yellow oil, which solidifies after cooling (49% yield).

2) Under argon atmosphere Mg turnings (1.22 g, 50.1 mmol) were suspended in dry THF (15 mL) and then 4-fluorobenzyl chloride (1.07 g, 7.4 mmol) was added in one portion. When the mixture started to become warm it was immediately cooled with an ice bath and then stirred for 2 h. After letting the residual Mg decanting, the supernatant was added dropwise to a solution of *N*-(*tert*-butoxy)-2-chloro-*N*-methylisonicotinamide (**29**, 900 mg, 3.7 mmol) in dry THF (10 mL), previously cooled at −10 °C. After completion of the addition the mixture was let slowly heating at rt and stirred for 3 h. NH_4_Cl saturated solution (80 mL) was added and the two formed phases were separated. The aqueous phase was then further extracted 3 times with EtOAc. The combined organic layers were then washed with NaCl saturated solution, dried over anhydrous Na_2_SO_4_, and concentrated at reduced pressure. Finally, the residue was purified by flash column chromatography (SiO_2_, *n*-hexane: EtOAc gradient elution from 4:1 to 3:2) giving 747 mg of pure product as a yellow oil, which solidifies after cooling (81% yield).

3) Under argon atmosphere Mg turnings (6.94 g, 285.6 mmol) were suspended in dry THF (35 mL). After that 4-fluorobenzyl chloride (13.6 g, 92.5 mmol) was added in portions: after adding 1 mL and starting the reaction, the mixture was cooled at 0 °C with an ice bath and the addition continued by 0.5 mL/min at the same temperature. After the addition was competed the mixture was let stirring until warming up to rt. After letting the Mg decanting the supernatant was added slowly dropwise to a suspension of 2-chloroisonicotinic acid (26, 6.0 g, 38.1 mmol) in dry THF (24 mL), previously cooled at −30 °C. During the addition the temperature was maintained between −25 and −30 °C. After complete addition the mixture was let heating to rt and stirred overnight. The mixture was poured on NH_4_Cl saturated solution (100 mL). The aqueous phase was extracted twice with EtOAc and the combined organic layers were dried over anhydrous Na_2_SO_4_, and concentrated at reduced pressure. Finally, the residue was purified by flash column chromatography (SiO_2_, petroleum ether 40/60:EtOAc gradient elution from 3:1 to 1:1) giving 3.37 g of pure compound as a yellow oil, which solidifies after cooling (35% yield); ^1^H-NMR (300 MHz, CDCl_3_) δ 4.24 (s, 2H), 6.99–7.10 (m, 2H), 7.15–7.23 (m, 2H), 7.67 (dd, *J* = 5.1, 1.5 Hz, 1H), 7.79 (dd, *J* = 1.4, 0.7 Hz, 1H), 8.56 (dd, *J* = 5.1, 0.5 Hz, 1H); ^13^C-NMR (75 MHz, CDCl_3_) δ 44.9, 115.9 (d, *J* = 21.6 Hz), 120.2, 122.7, 128.4 (d, *J* = 3.3 Hz), 131.1 (d, *J* = 8.3 Hz), 145.2, 151.0, 153.0, 162.2 (d, *J* = 246.6 Hz); ESI-MS: (*m*/*z)* 249.9 [M + H]^+^; HPLC: t_r_ = 5.555 min.

*1-(2-Chloropyridin-4-yl)-2-(4-fluorophenyl)-2-(hydroxyimino)ethan-1-one* (**30**). Ethanone derivative **25** (1.96 g, 7.85 mmol) was dissolved in glacial AcOH (15 mL) and after that NaNO_2_ (1.67 g, 24.2 mmol), previously dissolved in H_2_O (7 mL), was added dropwise and the reaction mixture was stirred overnight at rt. Afterwards 50 mL H_2_O were added and the voluminous precipitate formed was filtered off and washed with H_2_O. The residue was then suspended in H_2_O and the suspension was extracted with EtOAc. The combined organic layers were dried over anhydrous Na_2_SO_4_ and the solvent was then removed at reduced pressure, affording 1.8 g of the desired product, which was used for the following step without further purification (82% yield); ^1^H-NMR (300 MHz, DMSO-d_6_) δ 7.23–7.37 (m, 2H), 7.51–7.62 (m, 2H), 7.73 (dd, *J* = 5.0, 1.4 Hz, 1H), 7.89 (dd, *J* = 1.2, 0.7 Hz, 1H), 8.58 (dd, *J* = 5.0, 0.7 Hz, 1H), 13.14 (s, 1H); ^13^C-NMR (75 MHz, DMSO-*d*_6_) δ 115.3 (d, *J* = 21.6 Hz), 122.8, 124.3, 125.6 (d, *J* = 3.9 Hz), 132.5 (d, *J* = 8.3 Hz), 149.2, 150.5, 150.6, 154.3, 162.8 (d, *J* = 246.6 Hz), 190.4; ESI-MS: (*m*/*z)* 276.9 [M − H]^−^; HPLC: t_r_ = 7.405 min.

*1-(2-Chloropyridin-4-yl)-2-(4-fluorophenyl)ethan-1-one oxime* (**31**). Hydroxylamine hydrochloride (1.52 g, 21.93 mmol) was dissolved in H_2_O (3.5 mL) and then 20% NaOH_(aq)_ (0.7 mL) and a solution of ethanone derivative **25** (3.65 g, 14.62 mmol) in MeOH (17.5 mL) were added. The reaction mixture was stirred at rt for 48 h while a precipitate formed. Saturated NH_4_Cl solution (50 mL) was added and the precipitate was filtered off, rinsed with H_2_O and dried *in vacuo* over P_2_O_5_ affording 3.71 g of the desired product as an off-white solid, which was used for the following step without further purification (96% yield). Extraction with EtOAc represents an alternative work-up procedure giving similar yields; ^1^H-NMR (300 MHz, DMSO-*d*_6_) δ 4.16 (s, 2H), 7.12–7.02 (m, 2H), 7.28–7.20 (m, 2H), 7.64 (dd, *J* = 5.2, 1.5 Hz, 1H), 7.71–7.68 (m, 1H), 8.37 (d, *J* = 5.2 Hz, 1H), 12.27 (s, 1H); ^13^C-NMR (75 MHz, DMSO-*d*_6_) δ 29.0, 115.4 (d, *J* = 21.3 Hz), 119.7, 120.5, 130.2 (d, *J* = 8.0 Hz), 132.5 (d, *J* = 3.1 Hz), 146.5, 150.2, 151.0, 152.7, 160.9 (d, *J* = 242.4 Hz); ESI-MS: 265.0 [M + H]^+^, 263.0 [M − H]^−^; HPLC: t_r_ = 6.934 min.

*1-(2-Chloropyridin-4-yl)-2-(4-fluorophenyl)ethan-1-one O-tosyl oxime* (**32**). A solution of ketoxime 31 (1.20 g, 4.53 mmol) and *p*-toluenesulfonyl chloride (3.46 g, 18.14 mmol) in dry pyridine (6 mL) was stirred at rt under N_2_ atmosphere for 50 h. The mixture was poured into a NaCl saturated solution (30 mL) and extracted three times with EtOAc. The combined organic layers were then washed 3 times with a NaCl saturated solution, dried over anhydrous Na_2_SO_4_ and concentrated at reduced pressure. Finally, the residue was purified by flash column chromatography (SiO_2_, petroleum ether 40/60:EtOAc 2:3) giving 1.68 g of the desired product as a yellow oil (88% yield); ^1^H-NMR (300 MHz, CDCl_3_) δ 2.48 (s, 3H), 4.12 (s, 2H), 6.89–7.06 (m, 4H), 7.33 (dd, *J* = 5.2, 1.5 Hz, 1H), 7.39 (d, *J* = 8.2 Hz, 2H), 7.42–7.46 (m, 1H), 7.89 (d, *J* = 8.3 Hz, 2H), 8.39 (d, *J* = 5.2 Hz, 1H); ^13^C-NMR (75 MHz, CDCl_3_) δ 21.8, 32.6, 116.1 (d, *J* = 21.6 Hz), 120.0, 122.1, 128.8 (d, *J* = 3.3 Hz), 129.0, 129.9 (d, *J* = 7.7 Hz), 129.9, 131.9, 143.4, 145.9, 150.3, 152.4, 162.5 (d, *J* = 246.6 Hz), 161.5; ESI-MS: (*m*/*z)* 473.2 [M + Na + MeOH]^+^; HPLC: t_r_ = 9.342 min.

*2-Amino-1-(2-chloropyridin-4-yl)-2-(4-fluorophenyl)ethan-1-one hydrochloride* (**23**). In a three-necks round bottom flask under argon atmosphere K chunks (175 mg, 4.4 mmol) were added portionswise to EtOH_(abs)_ (20 mL). After complete dissolution the mixture was cooled at 0 °C and 1-(2-chloropyridin-4-yl)-2-(4-fluorophenyl)ethan-1-one *O*-tosyl oxime (**31**, 1.70 g, 4.0 mmol), previously dissolved in EtOH_(abs)_ (50 mL) was added slowly dropwise. The mixture was then stirred at 0 °C for 3 h and after that dry Et_2_O (250 mL) was added and the mixture was stirred for 30 min at rt. The white precipitate formed was removed by filtration and the filtrate was concentrated at reduced pressure. The residue was taken up in conc. HCl_(aq)_ and the mixture was stirred at 60 °C for 2 h. The residual solvent was removed at reduced pressure and the residue was treated with a mixture of THF:Et_2_O 1:2. The white precipitate obtained was filtered off and dried affording 725 mg of the desired product, which was used for the following step without further purification (60% yield); ESI-MS: (*m*/*z*) 264.9 [M + H]^+^, 263.0 [M − H]^−^; HPLC: t_r_ = 2.329 min.

*5-(2-Chloropyridin-4-yl)-4-(4-fluorophenyl)-1-(2-methoxyethyl)-1,3-dihydro-2H-imidazole-2-thione* (**22a**). The title compound was prepared following general procedure A starting from compound **23** (500 mg, 1.65 mmol) and 2-methoxyethyl isothiocyanate (**24a**, 965 mg, 8.25 mmol); The residue was treated with Et_2_O obtaining a precipitate, which was filtered off and dried. The filtrate was then concentrated at reduced pressure and the residue was purified by flash column chromatography (SiO_2_, *n*-hexane:EtOAc gradient elution from 7:3 to 1:1). 293 mg of the desired product were obtained in total (49% yield); ^1^H-NMR (300 MHz, DMSO-*d*_6_) δ 3.06 (s, 3H), 3.52–3.60 (m, 2H), 3.99–4.14 (m, 2H), 7.08–7.32 (m, 4H), 7.42 (d, *J* = 4.6 Hz, 1H), 7.64 (s, 1H), 8.49 (d, *J* = 4.9 Hz, 1H), 13.07 (br. s, 1H); ESI-MS: (*m*/*z*) 364.2 [M + H]^+^, 362.2 [M − H]^−^; HPLC: t_r_ = 5.321 min.

*5-(2-Chloropyridin-4-yl)-4-(4-fluorophenyl)-1-methyl-1,3-dihydro-2H-imidazole-2-thione* (**22b**). The title compound was prepared following general procedure A starting from compound **23** (400 mg, 1.32 mmol) and methyl isothiocyanate (**24b**, 643 mg, 6.62 mmol); After the addition of NaHCO_3_ saturated solution, a precipitate was formed, which was filtered off and purified twice by flash column chromatography (SiO_2_, DCM:EtOH gradient elution from 100:0 to 97:03) and (SiO_2_, DCM:EtOH 99:01). The obtained solid was treated with a mixture of Et_2_O:*n*-hexane 1:2 and the yellow precipitate obtained was then filtered off and dried, affording 255 mg of the desired compound (60% yield); ^1^H-NMR (300 MHz, DMSO-*d*_6_) δ 3.43 (s, 3H), 7.16–7.25 (m, 2H), 7.26–7.35 (m, 2H), 7.42 (dd, *J* = 5.1, 1.3 Hz, 1H), 7.6 (s, 1H), 8.49 (d, *J* = 5.0 Hz, 1H), 13.01 (br. s, 1H); ^13^C-NMR (75 MHz, DMSO-*d*_6_) δ 32.1, 115.9 (d, *J* = 22.1 Hz), 123.1, 123.9 (d, *J* = 2.8 Hz), 124.3, 125.1, 125.6, 129.7 (d, *J* = 8.3 Hz), 139.9, 150.7, 151.0, 161.9 (d, *J* = 246.6 Hz), 162.7; ESI-MS: (*m*/*z*) 318.3 [M − H]^−^; HPLC: t_r_ = 4.389 min.

*5-(2-Chloropyridin-4-yl)-4-(4-fluorophenyl)-1-phenyl-1,3-dihydro-2H-imidazole-2-thione* (**22c**). The title compound was prepared following general procedure A starting from compound **23** (400 mg, 1.32 mmol) and phenyl isothiocyanate (**24c**, 535 mg, 3.96 mmol); The residue was purified by flash column chromatography (SiO_2_, DCM:EtOH gradient elution from 100:0 to 98:02). The solid obtained was treated with a mixture of Et_2_O:*n*-hexane 1:2 and the white precipitate formed was then filtered off and dried, affording 260 mg of the desired compound (51% yield);^1^H-NMR (300 MHz, CDCl_3_) δ 6.69 (d, *J* = 4.7 Hz, 1H), 6.81 (s, 1H), 6.89–7.03 (m, 2H), 7.11–7.21 (m, 2H), 7.21–7.30 (m, 2H), 7.32–7.46 (m, 3H), 8.11 (d, *J* = 5.2 Hz, 1H), 12.63 (br. s, 1H); ESI-MS: (*m*/*z*) 380.5 [M − H]^−^; HPLC: t_r_ = 5.867 min.

*2-Chloro-4-(4-(4-fluorophenyl)-1-(2-methoxyethyl)-2-(methylthio)-1H-imidazol-5-yl)pyridine* (**21a**). The title compound was prepared following general procedure B starting from compound **22a** (263 mg, 0.72 mmol), *t*-BuONa (83 mg, 0.86 mmol), and iodomethane (511 mg, 3.6 mmol) obtaining 270 mg of the desired product, which was used for the following step without further purification (99% yield); ^1^H-NMR (300 MHz, CDCl_3_) δ 2.73 (s, 3H), 3.25 (s, 3H), 3.55 (t, *J* = 5.5 Hz, 2H), 4.00 (t, *J* = 5.5 Hz, 2H), 6.87–6.98 (m, 2H), 7.21 (dd, *J* = 5.1, 1.4 Hz, 1H), 7.33–7.41 (m, 2H), 7.42 (dd, *J* = 1.3, 0.6 Hz, 1H), 8.40 (dd, *J* = 5.1, 0.6 Hz, 1H); ^13^C-NMR (75 MHz, CDCl_3_) δ 16.0, 44.4, 58.9, 70.5, 115.3 (d, *J* = 21.6 Hz), 124.1, 125.7, 126.3, 128.9 (d, *J* = 8.3 Hz), 129.5 (d, *J* = 3.3 Hz), 139.5, 141.9, 145.4, 150.1, 152.1, 162.0 (d, *J* = 246.6 Hz); ESI-MS: (*m*/*z*) 378.3 [M + H]^+^; HPLC: t_r_ = 7.524 min.

*2-Chloro-4-(4-(4-fluorophenyl)-1-methyl-2-(methylthio)-1H-imidazol-5-yl)pyridine* (**21b**). The title compound was prepared following general procedure B starting from compound **22b** (225 mg, 0.70 mmol), *t*-BuONa (81 mg, 0.84 mmol), and iodomethane (497 mg, 3.5 mmol). The crude product was purified by flash column chromatography (SiO_2_, DCM:EtOH gradient elution from 100:0 to 99:01) giving 189 mg of the desired compound (80% yield); ^1^H-NMR (300 MHz, CDCl_3_) δ 2.73 (s, 3H), 3.49 (s, 3H), 6.90–7.02 (m, 2H), 7.13 (dd, *J* = 5.1, 1.4 Hz, 1H), 7.34–7.45 (m, 2H), 8.42 (d, *J* = 5.1 Hz, 1H); ^13^C-NMR (75 MHz, CDCl_3_) δ 15.8, 32.0, 115.4 (d, *J* = 21.6 Hz), 123.4, 124.8, 126.1, 129.1 (d, *J* = 7.7 Hz), 129.5 (d, *J* = 3.3 Hz), 139.8, 141.6, 146.1, 150.4, 152.4, 162.2 (d, *J* = 246.6 Hz); ESI-MS: (*m*/*z*) 334.3 [M + H]^+^; HPLC: t_r_ = 6.695 min.

*2-Chloro-4-(4-(4-fluorophenyl)-2-(methylthio)-1-phenyl-1H-imidazol-5-yl)pyridine* (**21c**). The title compound was prepared following general procedure B starting from compound **22c** (219 mg, 0.57 mmol), *t*-BuONa (55 mg, 0.57 mmol), and iodomethane (404 mg, 2.85 mmol). After adding H_2_O a precipitate was formed, which was filtered off and dried, giving 185 mg of the desired product that was used for the following step without further purification (82% yield); ^1^H-NMR (300 MHz, CDCl_3_) δ 2.68 (s, 3H), 6.82 (d, *J* = 4.7 Hz, 1H), 6.93 (s, 1H), 6.96–7.09 (m, 2H), 7.11–7.25 (m, 2H), 7.37–7.58 (m, 5H), 8.15 (d, *J* = 4.9 Hz, 1H); ^13^C-NMR (75 MHz, CDCl_3_) δ 15.1, 115.5 (d, *J* = 21.6 Hz), 122.6, 124.2, 126.2, 127.6, 129.5 (d, *J* = 3.3 Hz), 129.6, 129.7, 135.1, 140.8 (d, *J* = 7.7 Hz), 147.6, 149.6, 151.7, 162.4 (d, *J* = 247.1 Hz); ESI-MS: (*m*/*z*) 396.6 [M + H]^+^; HPLC: t_r_ = 9.718 min.

*N-(4-(4-(4-Fluorophenyl)-1-(2-methoxyethyl)-2-(methylthio)-1H-imidazol-5-yl)pyridin-2-yl)-3-(4-methoxyphenyl)propanamide* (**3a**) [14]. The title compound was prepared according to general procedure C starting from imidazole **21a** (100 mg, 0.26 mmol), 3-(4-methoxyphenyl)propanamide (72 mg, 0.40 mmol), Pd_2_(dba)_3_ (12 mg, 0.013 mmol), XantPhos (15 mg, 0.026 mmol), and Cs_2_CO_3_ (254 mg, 0.78 mmol). The residue was purified twice by flash column chromatography (SiO_2_, DCM:EtOH gradient elution from 100:0 to 95:05) and (SiO_2_, DCM:EtOH gradient elution from 100:0 to 97:03) giving 50 mg of the desired product (37% yield); ^1^H-NMR (300 MHz, CDCl_3_) δ 2.64–2.77 (m, 5H), 3.00 (t, *J* = 7.6 Hz, 2H), 3.24 (s, 3H), 3.51 (t, *J* = 6.0 Hz, 2H), 3.79 (s, 3H), 4.10 (t, *J* = 6.0 Hz, 2H), 6.79–6.88 (m, 2H), 6.88–6.99 (m, 3H), 7.11–7.20 (m, 2H), 7.36–7.47 (m, 2H), 8.16 (br. s, 1H), 8.24 (d, *J* = 5.1 Hz, 1H), 8.29 (s, 1H); ESI-MS: (*m*/*z*) 521.5 [M + H]^+^, 543.4 [M + Na]^+^, 519.5 [M − H]^−^; HPLC: t_r_ = 8.476 min.

*N-(4-(4-(4-Fluorophenyl)-1-methyl-2-(methylthio)-1H-imidazol-5-yl)pyridin-2-yl)-3-(4-methoxyphenyl)-propanamide* (**3b**). The title compound was prepared according to general procedure C starting from imidazole **21b** (70 mg, 0.21 mmol), 3-(4-methoxyphenyl)propanamide (75 mg, 0.41 mmol), Pd_2_(dba)_3_ (10 mg, 0.01 mmol), XantPhos (12 mg, 0.021 mmol), and Cs_2_CO_3_ (205 mg, 0.63 mmol). The residue was purified twice by flash column chromatography (SiO_2_, DCM:EtOH gradient elution from 99:01 to 95:05) and (SiO_2_, *n*-hexane:EtOAc gradient elution from 4:1 to 1:1) giving 44 mg of the desired product (45% yield); ^1^H-NMR (300 MHz, CDCl_3_) δ 2.62–2.81 (m, 5H), 3.00 (t, *J* = 7.5 Hz, 2H), 3.54 (s, 3H), 3.79 (s, 3H), 6.84 (d, *J* = 8.6 Hz, 2H), 6.88–7.00 (m, 3H), 7.14 (d, *J* = 8.6 Hz, 2H), 7.36–7.50 (m, 2H), 8.21 (d, *J* = 5.1 Hz, 1H), 8.27 (s, 1H), 8.36 (br. s, 1H); ^13^C-NMR (75 MHz, CDCl_3_) δ 16.1, 30.3, 32.1, 39.6, 55.3, 114.0, 114.6, 115.3 (d, *J* = 21.6 Hz), 121.1, 127.6, 129.1 (d, *J* = 7.7 Hz), 129.3, 130.0 (d, *J* = 3.3 Hz), 132.3, 139.3, 141.1, 145.3, 148.1, 151.9, 158.2, 162.1 (d, *J* = 246.6 Hz), 171.0; ESI-TOF-HRMS: (*m*/*z*) [M + H]^+^ calcd. for C_26_H_25_FN_4_O_2_S 477.1755, found 477.1762; HPLC: t_r_ = 8.855 min.

*N-(4-(4-(4-Fluorophenyl)-2-(methylthio)-1-phenyl-1H-imidazol-5-yl)pyridin-2-yl)-3-(4-methoxyphenyl)-propanamide* (**3c**). The title compound was prepared according to general procedure C starting from imidazole **21c** (70 mg, 0.18 mmol), 3-(4-methoxyphenyl)propanamide (64 mg, 0.36 mmol), Pd_2_(dba)_3_ (8 mg, 0.009 mmol), XantPhos (10 mg, 0.018 mmol), and Cs_2_CO_3_ (173 mg, 0.53 mmol). The residue was purified twice by flash column chromatography (SiO_2_, DCM:EtOH gradient elution from 99:01 to 95:05) and (SiO_2_, *n*-hexane:EtOAc gradient elution from 4:1 to 1:1) giving 24 mg of the desired product (25% yield); ^1^H-NMR (300 MHz, DMSO-*d*_6_) δ 2.54–2.68 (m, 5H), 2.70–2.84 (m, 2H), 3.71 (s, 3H), 6.77–6.90 (m, 3H), 7.05–7.22 (m, 4H), 7.25–7.36 (m, 2H), 7.39–7.60 (m, 5H), 7.93 (s, 1H), 8.16 (dd, *J* = 5.1, 0.7 Hz, 1H), 10.44 (s, 1H); ^13^C-NMR (75 MHz, CDCl_3_) δ 15.3, 30.3, 39.7, 55.2, 113.9, 114.5, 115.3 (d, *J* = 21.6 Hz), 120.5, 127.9, 129.1, 129.2, 129.4, 129.5 (d, *J* = 7.7 Hz), 129.8 (d, *J* = 2.8 Hz), 132.3, 135.4, 140.0, 140.8, 146.8, 147.2, 151.4, 158.1, 162.2 (d, *J* = 246.6 Hz), 170.4; ESI-TOF-HRMS: (*m*/*z*) [M + H]^+^ calcd. for C_31_H_27_FN_4_O_2_S 539.1911, found 539.1915; HPLC: t_r_ = 10.160 min.

*(S)-4-(4-(4-Fluorophenyl)-1-(2-methoxyethyl)-2-(methylthio)-1H-imidazol-5-yl)-N-(1-phenylethyl)pyridin-2-amine* (**4a**) [15]. The title compound was prepared according to general procedure D starting from compound **21a** (100 mg, 0.26 mmol). The reaction was stopped after 24 h. The residue was then purified twice by flash column chromatography (SiO_2_, DCM:EtOH gradient elution from 100:0 to 95:05) and (SiO_2_, DCM:EtOH gradient elution from 99:01 to 97:03) giving 50 mg of the desired product (41% yield); ^1^H-NMR (300 MHz, DMSO-*d*_6_) δ 1.42 (d, *J* = 6.8 Hz, 3H), 2.63 (s, 3H), 3.06 (s, 3H), 3.22–3.32 (m, 2H, partially overlapping with H_2_O signal), 3.78–3.95 (m, 2H), 4.90–5.09 (m, 1H), 6.35–6.52 (m, 2H), 7.01–7.13 (m, 2H), 7.13–7.24 (m, 2H), 7.24–7.46 (m, 6H), 8.03 (d, *J* = 5.1 Hz, 1H); ESI-MS: (*m*/*z*) 463.5 [M + H]^+^, 461.5 [M − H]^−^; HPLC: t_r_ = 6.398 min.

*(S)-4-(4-(4-Fluorophenyl)-1-methyl-2-(methylthio)-1H-imidazol-5-yl)-N-(1-phenylethyl)pyridin-2-amine* (**4b**) [15]. The title compound was prepared according to general procedure D starting from compound **21b** (35 mg, 0.105 mmol). The reaction was stopped after 32 h although not completed. The residue was then purified by preparative TLC (SiO_2_, DCM:EtOH 95:05) and by flash column chromatography (SiO_2_, *n*-hexane:EtOAc gradient elution from 7:3 to 1:1) giving 17 mg of the desired product (39% yield); ^1^H-NMR (300 MHz, CDCl_3_) δ 1.54 (d, *J* = 6.7 Hz, 3H), 2.64 (s, 3H), 3.08 (s, 3H), 4.47–4.69 (m, 1H), 5.51 (br. s, 1H), 6.05 (s, 1H), 6.42 (d, *J* = 4.7 Hz, 1 H), 6.76–6.95 (m, 2H), 7.14–7.53 (m, 7H, overlapping with the solvent peak), 8.05 (d, *J* = 5.1 Hz, 1 H); ESI-MS: (*m*/*z*) 419.3 [M + H]^+^, 417.2 [M − H]^−^; HPLC: t_r_ = 7.063 min.

*(S)-4-(4-(4-Fluorophenyl)-2-(methylthio)-1-phenyl-1H-imidazol-5-yl)-N-(1-phenylethyl)pyridin-2-amine* (**4c**). The title compound was prepared according to general procedure D starting from compound **21b** (42 mg, 0.106 mmol). The reaction was stopped after 26 h. The residue was purified by flash column chromatography (SiO_2_, *n*-hexane:EtOAc gradient elution from 9:1 to 1:1) giving 42 mg of the desired product (82% yield); ^1^H-NMR (300 MHz, CDCl_3_) δ 1.41 (d, *J* = 6.8 Hz, 3H), 2.63 (s, 3H), 4.16–4.34 (m, 1H), 5.18–5.36 (m, 1H), 5.90 (s, 1H), 6.21 (dd, *J* = 5.3, 1.2 Hz, 1H), 6.86–6.98 (m, 2H), 7.00–7.10 (m, 2H), 7.11–7.31 (m, 5H, overlapping with the solvent peak), 7.31–7.41 (m, 3H), 7.41–7.51 (m, 2H), 7.83 (d, *J* = 5.2 Hz, 1H); ^13^C-NMR (75 MHz, CDCl_3_) δ 15.3, 24.4, 52.2, 107.4, 114.1, 115.2 (d, *J* = 21.6 Hz), 125.6, 127.1, 127.6, 128.4, 128.7, 128.9, 129.2 (d, *J* = 7.7 Hz), 129.3, 129.9 (d, *J* = 3.3 Hz), 135.5, 139.2, 139.8, 144.0, 146.0, 147.4, 157.8, 162.0 (d, *J* = 246.0 Hz); ESI-TOF-HRMS: (*m*/*z*) [M + H]^+^ calcd. for C_29_H_25_FN_4_S 481.1857, found 481.1865; HPLC: t_r_ = 9.148 min.

*2-Amino-2-(2-chloropyridin-4-yl)-1-(4-fluorophenyl)ethan-1-one hydrochloride* (**35**) [13,30]. Oxime **34** [13,30] (500 mg, 1.8 mmol) and 10% Pd on charcoal (100 mg, 0.095 mmol) were placed in a Schlenk reaction tube, which was then evacuated and filled with a H_2_ atmosphere. Isopropanolic HCl (10 mL) was then added and the mixture was vigorously stirred at rt under a constant supply of H_2_ for 1 h. The solvent was evaporated at reduced pressure and then MeOH was added. The Pd catalyst was removed by filtration and the filtrate was concentrated at reduced pressure. Finally, the residue was treated with EtOAc and the white solid obtained was filtered off and dried, affording 520 mg of the desired product, which were used for the following step without further purification (92% yield); ESI-MS: (*m*/*z*) 264.9 [M + H]^+^, 262.9 [M − H]^−^; HPLC: t_r_ = 2.486 min.

*4-(2-Chloropyridin-4-yl)-5-(4-fluorophenyl)-1-methyl-1,3-dihydro-2H-imidazole-2-thione* (**36**). The title compound was prepared following general procedure A starting from compound **35** (550 mg, 1.82 mmol) and methyl isothiocyanate (**24b**, 665 mg, 9.1 mmol); the residue was then purified by flash column chromatography (SiO_2_, DCM:EtOH gradient elution from 100:0 to 95:05) obtaining 267 mg of the desired product (46% yield); ^1^H-NMR (300 MHz, DMSO-*d*_6_) δ 3.27 (s, 3H), 7.03 (dd, *J* = 5.3, 1.6 Hz, 1H), 7.32 (d, *J* = 1.1 Hz, 1H), 7.37–7.48 (m, 2H), 7.51–7.63 (m, 2H), 8.22 (d, *J* = 5.3 Hz, 1H), 13.09 (br. s, 1H); ESI-MS: (*m*/*z*) 318.0 [M − H]^−^; HPLC: t_r_ = 5.864 min.

*2-Chloro-4-(5-(4-fluorophenyl)-1-methyl-2-(methylthio)-1H-imidazol-4-yl)pyridine* (**37**). The title compound was prepared following general procedure B starting from compound **36** (267 mg, 0.83 mmol), *t*-BuONa (96.1 mg, 1.0 mmol), and iodomethane (639 mg, 4.15 mmol) obtaining 255 mg of the desired product, which was used for the following step without further purification (92% yield); ^1^H-NMR (300 MHz, CDCl_3_) δ 2.65 (s, 3H), 3.28 (s, 3H), 7.03 (d, *J* = 5.0 Hz, 1H), 7.08–7.31 (m, 4H), 7.44 (s, 1H), 8.02 (d, *J* = 5.2 Hz, 1H); ^13^C-NMR (75 MHz, CDCl_3_) δ 15.6, 31.3, 116.7 (d, *J* = 21.6 Hz), 118.7, 120.4, 125.6 (d, *J* = 3.3 Hz), 132.3 (d, *J* = 8.3 Hz), 132.5, 134.3, 144.7, 144.9, 149.2, 151.7, 163.3 (d, *J* = 251.0 Hz); ESI-MS: (*m*/*z*) 334.0 [M + H]^+^; HPLC: t_r_ = 8.065 min.

*N-(4-(5-(4-Fluorophenyl)-1-methyl-2-(methylthio)-1H-imidazol-4-yl)pyridin-2-yl)-3-(4-methoxyphenyl)-propanamide* (**38**). The title compound was prepared according to general procedure C starting from imidazole **37** (90 mg, 0.27 mmol), 3-(4-methoxyphenyl)propanamide (73 mg, 0.41 mmol), Pd_2_(dba)_3_ (12 mg, 0.013 mmol), XantPhos (16 mg, 0.027 mmol), and Cs_2_CO_3_ (264 mg, 0.81 mmol). The residue was purified by flash column chromatography (SiO_2_, DCM:EtOH gradient elution from 100:0 to 95:05) affording 82 mg of the desired product (64% yield); ^1^H-NMR (300 MHz, CDCl_3_) δ 2.59 (t, *J* = 7.7 Hz, 2H), 2.76 (s, 3H), 2.94 (t, *J* = 7.6 Hz, 2H), 3.37 (s, 3H), 3.78 (s, 3H), 6.82 (d, *J* = 8.6 Hz, 2H), 7.05–7.15 (m, 3H), 7.18–7.27 (m, 2H), 7.29–7.38 (m, 2H), 8.00 (d, *J* = 5.4 Hz, 1H), 8.21–8.42 (m, 2H); ^13^C-NMR (75 MHz, CDCl_3_) δ 15.7, 30.3, 31.3, 39.6, 55.2, 110.8, 113.9, 116.5 (d, *J* = 21.6 Hz), 116.8, 125.9 (d, *J* = 3.3 Hz), 129.3, 132.2, 132.4 (d, *J* = 8.3 Hz), 132.6, 135.7, 144.4, 144.5, 146.9, 151.5, 158.0, 163.3 (d, *J* = 246.6 Hz), 170.4; ESI-TOF-HRMS: (*m*/*z*) [M + H]^+^ calcd. for C_26_H_25_FN_4_O_2_S 477.1755, found 477.1763; HPLC: t_r_ = 7.745 min.

*(S)-4-(5-(4-Fluorophenyl)-1-methyl-2-(methylthio)-1H-imidazol-4-yl)-N-(1-phenylethyl)pyridin-2-amine* (**39**). The title compound was prepared according to general procedure D starting from compound **21a** (55 mg, 0.16 mmol). The reaction was stopped after 40 h although not fully completed. The residue was then purified by flash column chromatography (SiO_2_, *n*-hexane:EtOAc gradient elution from 4:1 to 1:1) obtaining 39 mg of the desired product (58% yield); ^1^H-NMR (300 MHz, CDCl_3_) δ 1.39 (d, *J* = 6.8 Hz, 3H), 2.60 (s, 3H), 3.22 (s, 3H), 4.37–4.50 (m, 1 H), 4.91 (d, *J* = 6.4 Hz, 1H), 6.30 (s, 1H), 6.57 (dd, *J* = 5.5, 1.4 Hz, 1H), 6.98–7.31 (m, 9H overlapping with the solvent peak), 7.80 (d, *J* = 5.4 Hz, 1H); ^13^C-NMR (75 MHz, CDCl_3_) δ 15.8, 24.2, 31.2, 51.7, 103.4, 110.9, 116.3 (d, *J* = 21.6 Hz), 125.8, 126.3 (d, *J* = 3.9 Hz), 126.8, 128.4, 131.3, 132.4 (d, *J* = 8.3 Hz), 136.1, 143.1, 143.8, 144.6, 147.6, 158.0, 163.0 (d, *J* = 249.9 Hz); ESI-TOF-HRMS: (*m*/*z*) [M + H]^+^ calcd. for C_24_H_23_FN_4_S 419.1700, found 419.1706; HPLC: t_r_ = 6.899 min.

## Figures and Tables

**Table 1:** Biological activity of tetrasubstituted pyridinylimidazoles **3a**–**c**, **4a**–**c**, **38**, and **39**.

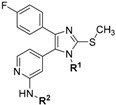			
**Cpd.**	**R^1^**	**R^2^**	**IC_50_** (μM) Mean ± **SEM ^a^**
**3a**	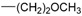	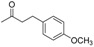	0.048 ± 0.010 ^b^
**3b**	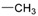	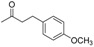	0.020 ± 0.004
**3c**	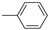	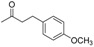	0.413 ± 0.072
**4a**	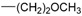	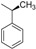	0.040 ± 0.010 ^c^
**4b**	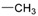	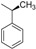	0.070 ± 0.030 ^c^
**4c**	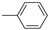	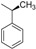	1.51 ± 0.15
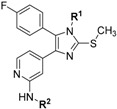			
**Cpd.**	**R^1^**	**R^2^**	**IC_50_ (μM)** **Mean ± SEM ^a^**
**38**	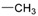	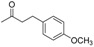	2.70 ± 0.02
**39**	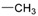	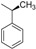	3.19 ± 0.78

^a^ *n* = 3; ^b^ data taken from ref. [14]; ^c^ data taken from ref. [15], *n* = 4.
